# Correction: Predicting direct and indirect non-target impacts of biocontrol agents using machine-learning approaches

**DOI:** 10.1371/journal.pone.0258080

**Published:** 2021-09-29

**Authors:** Hannah J. Kotula, Guadalupe Peralta, Carol M. Frost, Jacqui H. Todd, Jason M. Tylianakis

Fig 3 is incorrect. Additionally, part of the caption for Fig 3 erroneously appeared in the Results section of the published article, specifically the “Can random forest and KNN predict interaction frequencies and does this predictive ability vary with species’ generality and abundance, and whether the model was trained on data from the same vs. different habitat?” subsection. The authors have provided a corrected version of Fig 3 and its full caption here.

**Fig 3:**
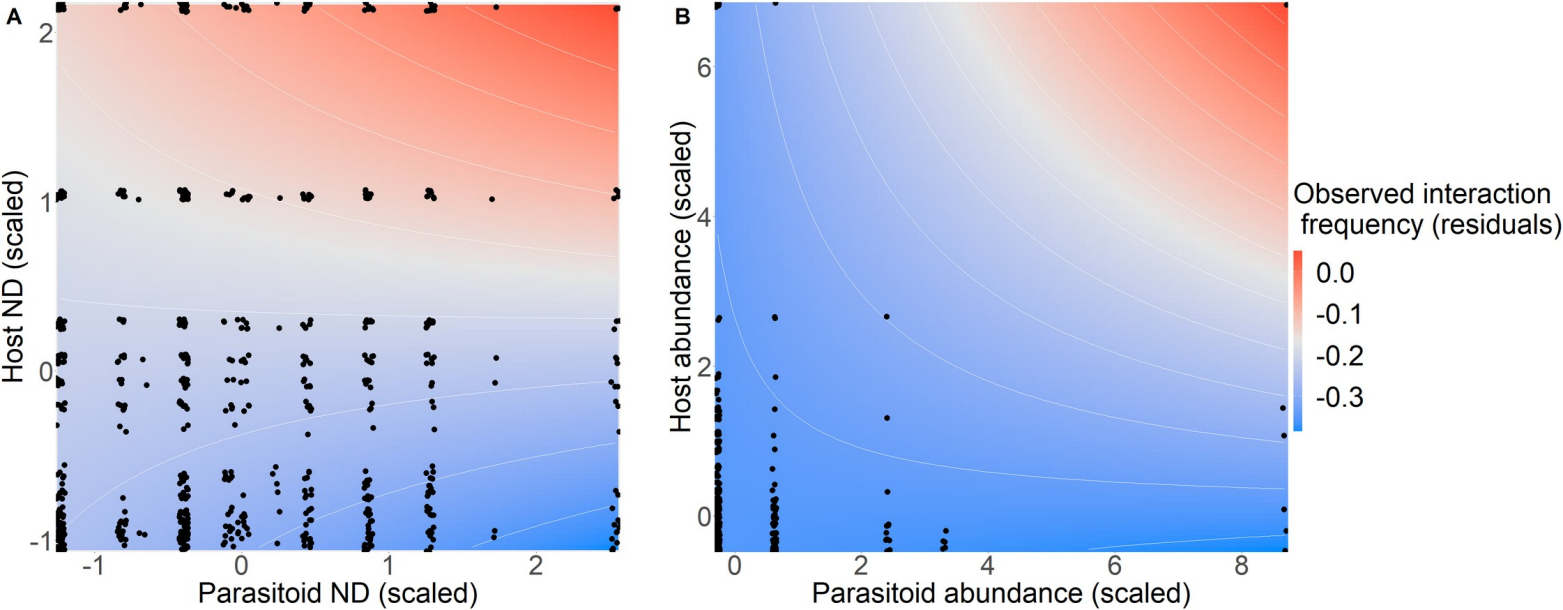
Overall, machine-learning predictions of observed interaction frequency varied with the generality (measured as normalised degree; ND) of the interacting partners for KNN, but not random-forest models, and depended on the abundance of the interacting partners for both models. There was a significant three-way interaction between predicted-frequency and generality of the interacting partners (host and parasitoid) in the KNN ‘native’ model. Additionally, there was a significant three-way interaction between predicted-frequency (or -probability) and abundance of the interacting partners for 4/6 models. Two are presented here to demonstrate the main patterns: (A) KNN ‘native’ model (which had a significant positive predicted frequency x host ND x parasitoid ND interaction), (B) random-forest ‘combined’ model, which had a significant host abundance x parasitoid abundance x predicted probability interaction (as did the ‘plantation’ random-forest model and the KNN ‘plantation’ and ‘combined’ models). However, the limited data between species with extreme abundance values, means this result should be treated with caution. Deviance residuals of the best-fitting model excluding the host and parasitoid ND (or abundance) variables are plotted as colour contours, to indicate whether observed interaction frequencies are higher (red) or lower (blue) than expected based on machine-learning predictions, showing how predictions vary with generality (or abundance) of the interacting partners. Each point represents a host-parasitoid species pair present within a site at the first-time step (*t*) test sites.
