# Supplementary figures and images for: Predicting direct and indirect non-target impacts of biocontrol agents using machine-learning approaches
Source: PLoS One. 2021 Jun 1;16(6):e0252448. doi: 10.1371/journal.pone.0252448 (PMC8168882; doi:10.1371/journal.pone.0252448)

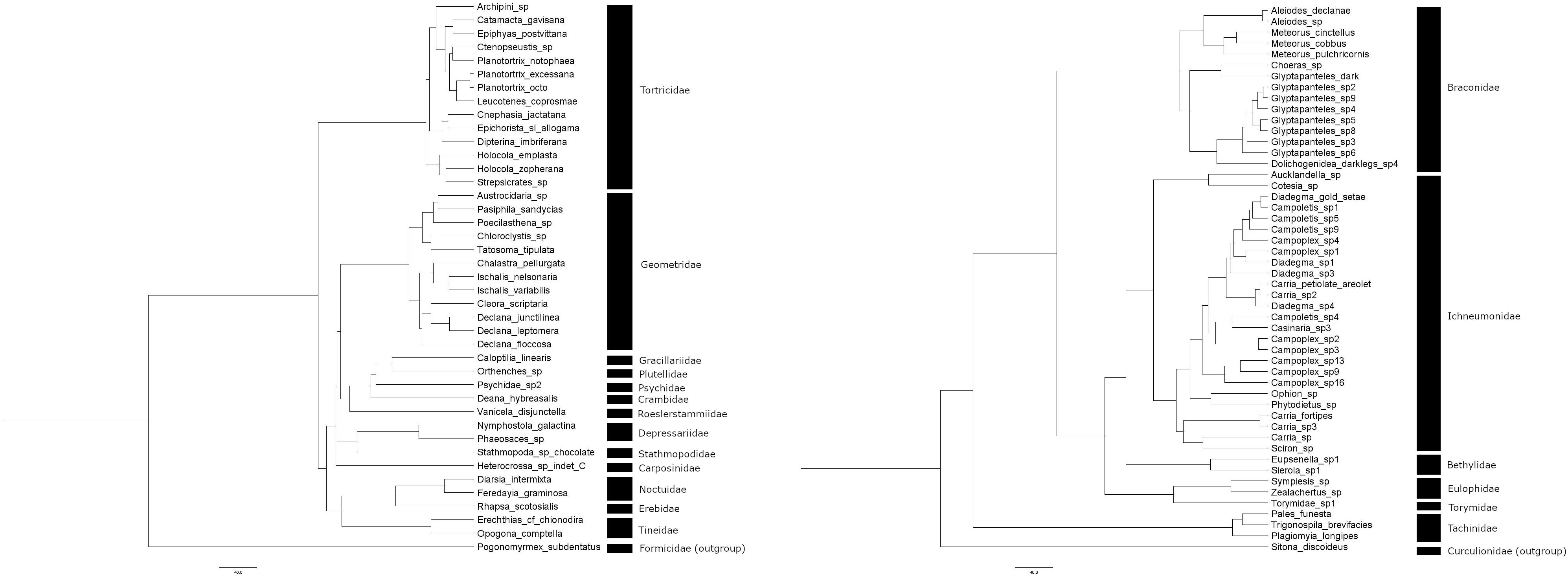

Supplement: S1 Fig — (TIF) [file pone.0252448.s008.tif]
