# Supplementary material for: Predicting direct and indirect non-target impacts of biocontrol agents using machine-learning approaches
Source: PLoS One. 2021 Jun 1;16(6):e0252448. doi: 10.1371/journal.pone.0252448 (PMC8168882; doi:10.1371/journal.pone.0252448)
Supplement: Attachment — Submitted filename: response_to_reviewers.docx [file pone.0252448.s010.docx]

**Kotula et al. Response to Reviewers**

10 May 2021

Dear Dr. Stephens,

On behalf of my co-authors, I would like to thank you and the two reviewers for the helpful comments on our manuscript. My co-authors and I appreciate the suggestions, and we present point-by-point responses (in blue text) to each comment below, along with tracked changes in the submitted manuscript and supporting information. This is the second review of our manuscript, and we feel that the reviewer’s comments have all been suitably addressed, and that the manuscript is improved. Below we also provide an updated funding statement. If you have any further questions, concerns, or revisions, please do not hesitate to contact us.

Kind regards,

Hannah Kotula, Guadalupe Peralta, Carol Frost, Jacqui Todd, and Jason Tylianakis

**Amended funding statement:**

H.J.K acknowledges support from a Roland Stead Postgraduate Scholarship in Biology. HJK, GP and JMT were funded by the Marsden Fund (grant number UOC1705), administered by the Royal Society of New Zealand. J.H.T is funded by the Better Border Biosecurity (B3) (www.b3nz.org) research collaboration funded by the New Zealand Government.

We acknowledge that one of the authors (J.H.T) is employed by a commercial company (The New Zealand Institute for Plant and Food Research Limited). Although this company does both government-funded and industry-funded work, there is no industry funding for this research. The funder provided support in the form of salaries for an author [J.H.T], but did not have any additional role in the study design, data collection and analysis, decision to publish, or preparation of the manuscript. The specific role of this author is articulated in the ‘author contributions’ section.

**Editor’s comments**

Thank you for submitting your manuscript to PLOS ONE. After careful consideration, we feel that it has merit but does not fully meet PLOS ONE’s publication criteria as it currently stands. Therefore, we invite you to submit a revised version of the manuscript that addresses the points raised during the review process.

You have done an excellent job responding to the first round of reviews. However, both reviewers found some minor issues that still need to be addressed.

There also may be some issue with the line numbers, the ones I was seeing in my draft obviously differed from those that reviewer 3 was looking at. For clarity, I am reiterating reviewer 3's concerns:

Thank you for your comments, and we apologise for the difference in line numbers. Thanks also for reiterating reviewer 3’s comments.

Line 357, which refers to equation one. K does not occur anywhere in equation 1.

Thanks for noticing that k doesn’t occur in equation 1, and we apologise for this mistake. We have now removed “*k* is a parasitoid species” from line 358.

Line 1007: I am not entirely sure what "error caused by gremlins" reviewer three is referring to. However, the journal capitalization on line 1007 is different from that of the rest of the references (only the first word is capitalized). In reference 83, there is also some unnecessary capitalization of the article title.

It would be a good idea to double check the formatting of the references before your final submission, it’s very easy for minor errors to creep into the sources cited.

Thanks for pointing this out. We have now double checked the formatting of the references for both the main text and the appendix.

As long as you can deal with the minor remaining issues raised by the reviewers, this should be ready to go. I leave it to your discretion whether to implement reviewer three's other suggestion for figure 1. It would enhance readability, but the figure seems fine in its current state to me (and a great improvement to this manuscript!).

Thanks for this. We agree and have kept the figure as it was (see response to reviewer 3’s suggestion for figure 1 below).

**Reviewer #2:** Dear Dr. Stephens, dear authors,

This is my second review of the manuscript ‘predicting direct and indirect non-targets of biocontrol agents using machine-learning approaches’ by Kotula et al. and it was a pleasure to review the manuscript again

The authors have addressed and fulfilled all the points raised by both of the reviewers: they added a conceptional figure explaining their approach, they used a blocked CV, they fitted the ML models without using the species abundances as predictors, and they admitted to make all code/methods freely available.

I think that the manuscript in its current form would make a great contribution to the community and fits perfectly into the scope of PLOS ONE.

I added below a few general and specific comments.

Thanks for your comments. Yes, our code is now available on GitHub (<https://github.com/hannah-jk/predicting_nontarget_impacts>), and we have now included this link in the main text (just below the acknowledgements section).

===== General comments =====

Check for consistency, sometimes the authors write ‘random forest’ and then ‘random-forest’.

Thanks for pointing this out. We have now double checked this and we now always use “random forest” (except in cases where it is used as a compound adjective, like “random-forest model” where we use a hyphen).

The authors said that they have now included the formulas for their mixed-effect models but I could not find them.

We have included the formulas for the mixed-effect models in Appendix S1 (Tables 21-24). We have now added “*The R formulas for these, and all subsequent, models are included in Appendix S1 (Tables 21-24)*” to line 414 to make this clear.

Optional: I think that the authors could make explicit connections between their research and its relevance for conservation and biodiversity ecology. For instance, the authors could expand their conclusion in the abstract which falls short compared to the rest of the abstract.

Thanks for the suggestion. The last sentence of the abstract now reads:

“*Combining machine-learning and network approaches provides a starting point for reducing risk in biocontrol introductions, and could be applied more generally to predicting species interactions such as impacts of invasive species*.”

===== Specific comments =====
L258: either you provide some references for the Netflix problem (e.g. https://peerj.com/articles/3644/) or you omit this sentence

We have now added the reference you suggest.

L306-308: Can you provide a reference for this statement?

Yes, we have now added a reference for this statement.

L404-405: scaling doesn’t result in a sd of 1?

We used the scale function from base R with centre=TRUE, scale=TRUE which scales variables so that they have a mean of zero and a standard deviation of one. We have added a few words to this sentence in the manuscript to make it more clear, and it now reads (L404-407):

“*For each model, we scaled and centred the fixed effect (by subtracting the mean and dividing by the standard deviation such that the scaled variable would have a mean of zero and a standard deviation of one) to help with model convergence*.”

L434: I don’t think this avoids the problem of stepwise selection of variables because the selection after the AIC is itself the problem (the problem is that the AIC selects the best predictive model and not the causal one (BIC does, but only approximatively)). Maybe just omit the part ‘thereby avoiding…’

We agree and we have now omitted “thereby avoiding issues associated with stepwise selection of variables.”

L702-717: Well, kNN is also a very ‘simple’ (less complex) ML algorithm compared to RF and kNN is also unable to infer automatically interactions between predictors and is less able to handle non-linearities

Thanks for this comment. We agree and we have now added (L719-722): “*Additionally, random forest is a more complex algorithm than KNN. Because random forest is based on many trees (each with many branches), random forest is able to account for interactions between variables and non-linearities. This may also have contributed to the better predictive ability of random forest compared to KNN*.”

**Reviewer #3:** The authors present a very convincing and appealing case for the use of ML to infer potential consequences of biocontrol agents looking at both direct and indirect effects, in addition to comparing two different ML approaches. After the initial round of review it seems that both reviewers were primarily concerned with the ‘density’ of the manuscript and (barring some smaller comments) were very convinced by the presentation of the statistical/analysis portion. I believe that the authors have taken the feedback from the initial reviewers to heart and that the inclusion of the conceptual figure (Fig. 1) as well as some of the rephrasing of sections has made the manuscript substantially easier to follow and will surely make it more accessible to a general audience. I think showcasing the two streams of direct and indirect effects and how/when data are shared in the conceptual figure has made the storyline much easier to follow.

I would like to commend the authors on managing to condense all this information to be contained within a single manuscript and I have one stylistic comment/query regarding the conceptual figure and one point with regards to equation 1 (l. 366) but otherwise believe that the manuscript complies to the PLOS one guidelines and should be considered ready for publication.

Thanks for your comments.

I wonder if it may be possible to add short subheadings along with the letters to make the figure more intuitive at face value without having to refer to the figure legend? You already have this for a few of the points _e.g._ g, e, i and it would be a case of bolding them and ‘marrying’ them to the letter. This feeling might be in part due to the submission requirements of the journal and having the legend split form the figure may have contributed to me feeling the need for some subheading in the figure. I do just want to stress that I don't think altering the figure is _critical_ run order for the manuscript to progress.

Thanks for your suggestion. We feel that the figure as it stands has about the right amount of text. When the figure and legend are together, we feel they clearly explain our methods.

Could the authors have a second look at the descriptive breakdown of equation 1? In line 266 the authors state that _k_ is a parasitoid species. However, there is no variable _k_ indicated in the actual equation.

Thanks for noticing this, and we apologise for this mistake. The editor reiterated this comment above, and we have responded to it there.
\## Minor comment

A gremlin crept into your reference list and tinkered with reference number 83 - line 1107

Thanks for noticing this. We have now double checked the reference list.
